# Checkpoint Inhibitor-Associated Myasthenia–Myositis Overlap With Neuromuscular Respiratory Failure After Dual PD-L1 Plus CTLA-4 Checkpoint Blockade for Hepatocellular Carcinoma

**DOI:** 10.7759/cureus.93341

**Published:** 2025-09-27

**Authors:** Adam M Bowen, Dania Baraka, Ramalakshmi Thulluri, Miller Lantis, Claire Russell, Taylor Patterson, Shannon Pierce, Alfarooq Alshaikhli

**Affiliations:** 1 Hematology and Oncology, Upstate University Hospital, Syracuse, USA; 2 Internal Medicine, McLaren Greater Lansing Hospital, Lansing, USA; 3 Hematology and Oncology, McLaren Greater Lansing Hospital, Lansing, USA

**Keywords:** ctla‑4 inhibitor, hepatocellular carcinoma, immune checkpoint inhibitor, immune‑related adverse event, myasthenia gravis, myositis, neuromuscular respiratory failure, overlap syndrome, pd‑l1 inhibitor

## Abstract

Immune checkpoint inhibitors are standard therapy for unresectable hepatocellular carcinoma (HCC), but rare immune-related adverse events may be life-threatening. We describe an 80-year-old man with unresectable HCC who received dual durvalumab and tremelimumab on a non-STRIDE schedule. Within four weeks, he developed ptosis, diplopia, and bulbar weakness that rapidly progressed to neuromuscular respiratory failure. Laboratory evaluation revealed markedly elevated creatine kinase, troponin, and aldolase levels, along with a strongly positive acetylcholine receptor antibody. Despite treatment with high-dose corticosteroids, intravenous immunoglobulin, and plasma exchange, neurologic recovery was limited, and he experienced recurrent respiratory decline. After confirming decision-making capacity, he chose to discontinue life-prolonging therapy and transitioned to hospice. This case highlights the fulminant potential of myasthenia-myositis overlap after programmed death-ligand 1 (PD-L1) plus cytotoxic T-lymphocyte-associated protein 4 (CTLA-4) blockade, underscores the importance of early biomarker surveillance and rapid escalation to immunomodulatory therapy, and illustrates the need for early goals-of-care discussions in patients facing severe immune-related toxicities.

## Introduction

The combination of checkpoint inhibition with durvalumab and tremelimumab has reshaped the treatment landscape for unresectable hepatocellular carcinoma (HCC). In the HIMALAYA program, the regimen known as STRIDE consisted of a single priming dose of tremelimumab combined with regular-interval durvalumab. It demonstrated an overall survival advantage compared with sorafenib [1,2]. Our patient did not receive STRIDE; instead, he was treated with durvalumab every four weeks, together with repeated tremelimumab dosing, a non-STRIDE schedule that still constitutes dual programmed death-ligand 1 (PD-L1) and cytotoxic T-lymphocyte-associated protein 4 (CTLA-4) blockade. While the HIMALAYA trial established efficacy for the STRIDE protocol, the safety profile of alternative dosing schedules remains less well defined.

Immune-related adverse events (irAEs) are toxicities that result from loss of self-tolerance after checkpoint blockade. These can affect almost any organ system, and while dermatologic and gastrointestinal irAEs are more common, neurologic irAEs are rarer but often fulminant and associated with high mortality.

Neurologic irAEs are uncommon but can be fulminant. Among these, a myasthenia-myositis-myocarditis (MMM) overlap syndrome has been increasingly recognized and carries significant in-hospital mortality [3]. Importantly, MMM overlap is best understood as a spectrum: some patients manifest all three features, while others present with only myasthenia and myositis or with myasthenia alone. Myocarditis may be possible but not always demonstrable, and careful correlation of clinical, serologic, and imaging evidence is required. Immune checkpoint inhibitor (ICI)-associated myasthenia gravis typically arises early after therapy initiation, progresses rapidly, and has higher mortality than idiopathic MG [4]. Red-flag features include ocular and bulbar weakness (ptosis, diplopia, dysphagia), evolving dyspnea due to diaphragmatic involvement, and elevations in creatine kinase (CK) and cardiac troponin that suggest concurrent myositis or myocarditis [3]. Current guidance recommends the immediate interruption of checkpoint inhibitors, initiation of high-dose corticosteroids, and rapid escalation to intravenous immunoglobulin (IVIG) and/or therapeutic plasma exchange (PLEX) for bulbar, respiratory, or cardiac involvement within a coordinated multidisciplinary approach [5,6].

This report presents a life-threatening MMM-overlap syndrome during repeated-dose tremelimumab plus durvalumab and underscores the need for early recognition and prompt management.

## Case presentation

**Figure 1:**
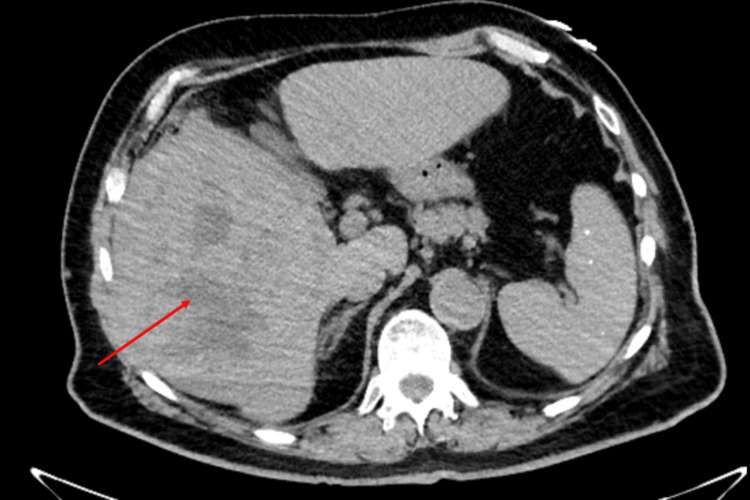
Axial CT of the abdomen showing a large right hepatic lobe mass (arrow) consistent with HCC CT: computed tomography, HCC: hepatocellular carcinoma

An 80-year-old man with insulin-dependent diabetes, hypertension, hyperlipidemia, prior tobacco use, and treated hepatitis C. Family history was negative for neuromuscular or autoimmune disease, and there was a history of a father with colon cancer and a brother with an unknown type of cancer. Baseline medications included amlodipine, lisinopril, hydrochlorothiazide, terazosin, simvastatin, aspirin, pantoprazole, ferrous sulfate, and multiple insulin preparations (neutral protamine hagedorn and regular). The patient presented with several months of right-sided epigastric discomfort, mild nausea, and an unintentional 20-pound (9-kg) weight loss. CT revealed a right hepatic mass, and subsequent contrast-enhanced MRI of the abdomen demonstrated a large heterogeneous lesion in the central to caudal right hepatic lobe measuring approximately 17 × 13 × 15 cm, with areas of probable necrosis, arterial-phase enhancement, and washout (Figure 1). A CT-guided biopsy showed atypical hepatocytic proliferation with mild to moderate cytologic atypia, consistent with HCC.

Immunohistochemistry demonstrated hepatocyte antigen positivity, accompanied by diffuse sinusoidal CD34 staining, while glypican-3 was negative. These findings were consistent with HCC. The serum alpha-fetoprotein was low at <3 ng/mL. Although alpha-fetoprotein is commonly used for HCC surveillance and diagnosis, it may be normal even in advanced tumors; thus, the diagnosis in this case relied primarily on imaging and histopathology. No other tumor markers (CEA, CA 19-9, PIVKA-II) were elevated or clinically contributory in this patient. Other laboratory studies at baseline, including complete blood count, metabolic panel, and coagulation profile, were unremarkable apart from elevated transaminases (ALT 206 U/L, AST 156 U/L). Given the large tumor size and comorbidities, he was deemed a nonsurgical candidate, and a multidisciplinary team recommended locoregional therapy with planned Y-90 in addition to combined checkpoint inhibition.

He initiated combined checkpoint inhibition with durvalumab 1500 mg IV every four weeks, along with tremelimumab 300 mg IV every four weeks, a repeated-dosing regimen that differed from the STRIDE protocol [1]. Approximately one month after his first cycle, he underwent interventional radiology mapping. Approximately one month after his first cycle, he underwent interventional radiology mapping in preparation for planned Y-90 radioembolization, which the multidisciplinary team had recommended to provide locoregional control of his large, unresectable hepatic tumor in the setting of limited systemic options. Within hours, he developed heaviness of the left eyelid and new horizontal diplopia, which improved with monocular viewing.

**Figure 2:**
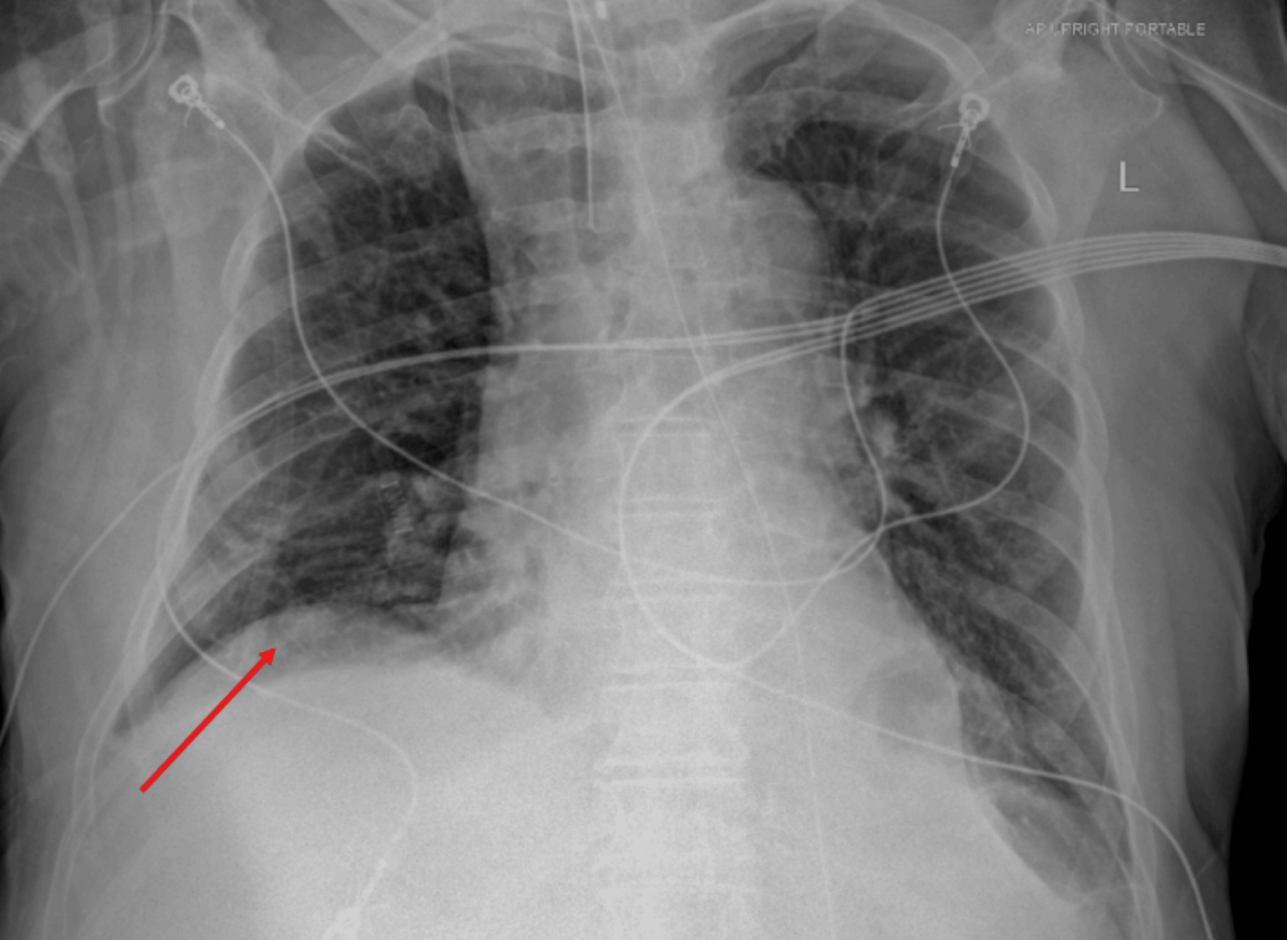
Portable chest radiograph demonstrating elevation of the right hemidiaphragm with adjacent basilar opacities (arrow) consistent with atelectasis or pneumonia

The next morning, following a brief episode of syncope, he presented to an outside emergency department. He was hypoxemic on arrival and tested positive for influenza A. He was started on oseltamivir 75 mg orally twice daily and completed a full five-day antiviral course during hospitalization. Chest radiography revealed bilateral hazy opacities and elevation of the right hemidiaphragm, consistent with viral pneumonia and possible atelectasis (Figure 2).

**Figure 3:**
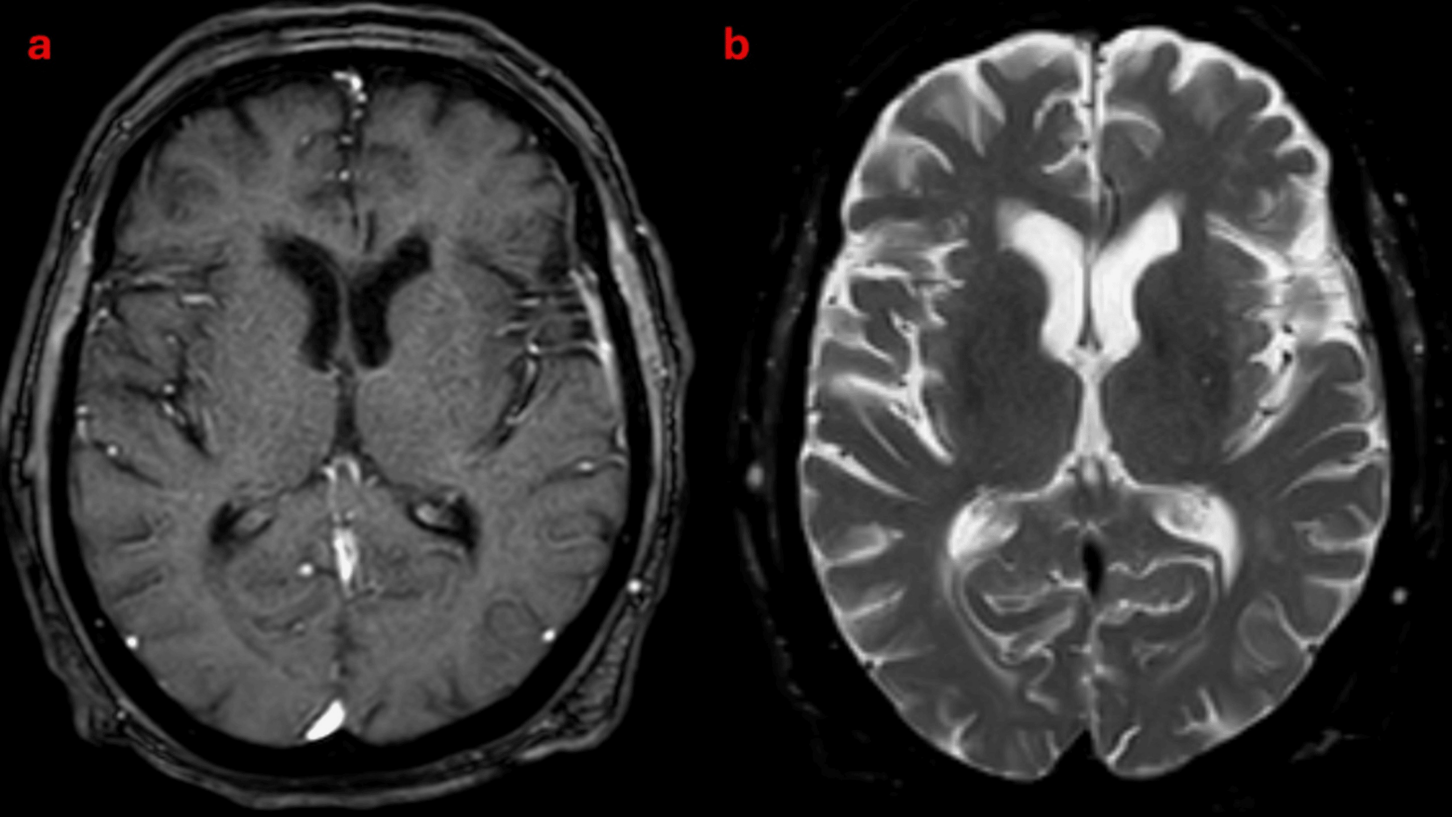
Axial brain MRI showing no acute intracranial lesion. (a) T1-weighted image and (b) T2-weighted image demonstrating preserved parenchymal architecture without acute ischemia or mass effect MRI: magnetic resonane imaging

Over the first 48 hours, his neurologic symptoms progressed. The left ptosis persisted, and he developed right facial droop, dysphagia, and difficulty managing secretions. Examination revealed severe limitation of horizontal eye movements with preserved vertical gaze, bilateral ptosis that fluctuated with fatigue, and right facial weakness. Peripheral vision and limb sensation were intact. Brain MRI did not show an acute lesion, and vascular studies suggested right vertebral artery abnormalities that did not explain the evolving ocular-bulbar pattern (Figure 3).

**Figure 4:**
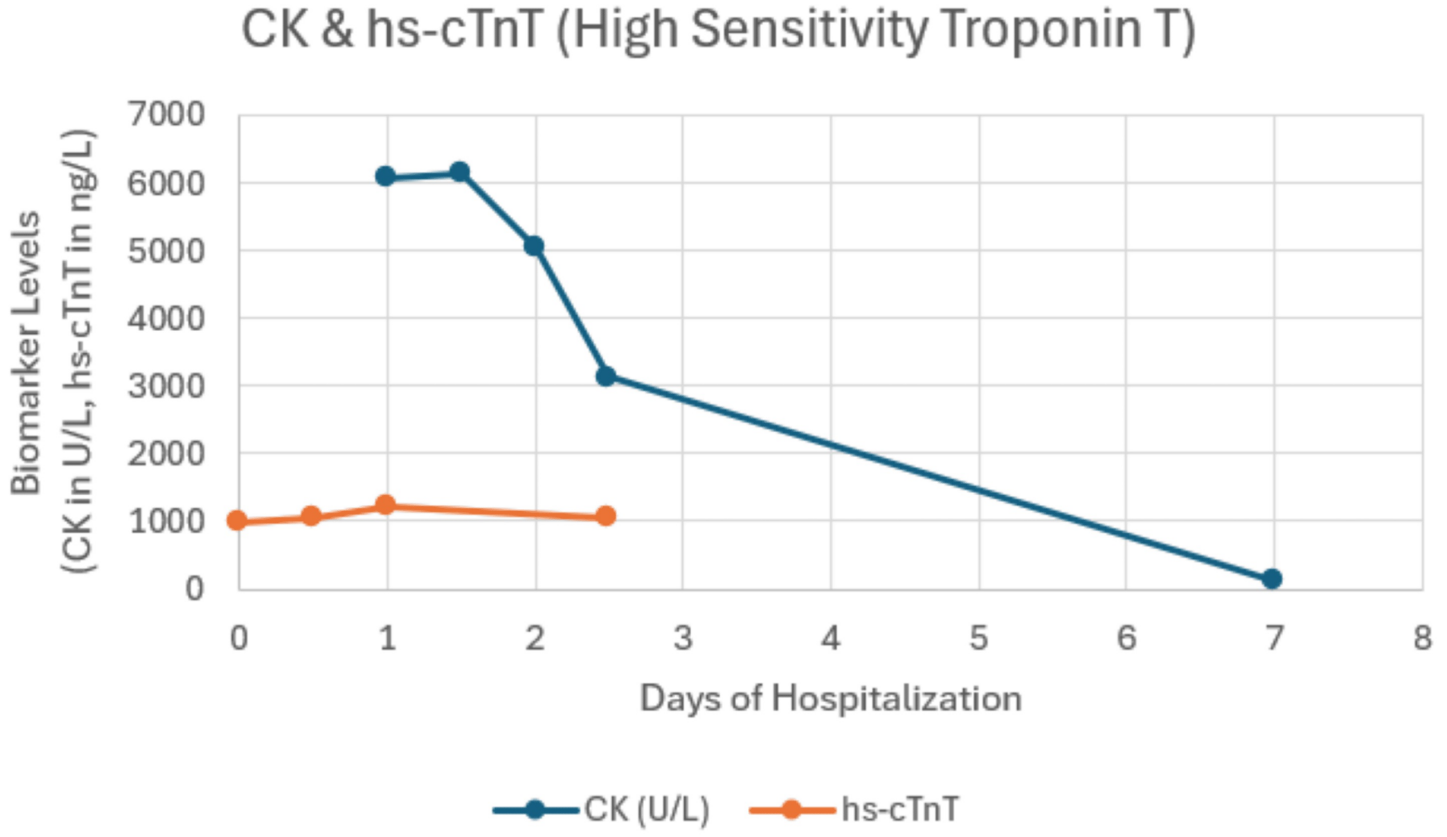
Trends in C and hs-cTnT levels during hospitalization demonstrating elevated biomarkers at presentation with gradual decline following immunomodulatory therapy CK: creatine kinase, hs-cTnT: high-sensitivity troponin T

On the day of ICU transfer, he developed rapidly worsening respiratory distress with accessory muscle use and required intubation for neuromuscular respiratory failure. Laboratory studies revealed a peak CK of 6,070 U/L, a peak high-sensitivity troponin of 1,214 ng/L, and an elevated aldolase of 64.6 U/L (Figure 4).

Cerebrospinal fluid analysis, performed on hospital day 2 after ICU transfer, showed protein at 46.3 mg/dL and glucose at 131 mg/dL, with only two nucleated cells and absent oligoclonal bands, findings not consistent with meningitis or Guillain-Barré syndrome (GBS). The acetylcholine receptor (AChR) antibody was strongly positive at 27.9 nmol/L, while MuSK and ganglioside antibodies (GM1, GQ1b) were negative. Additional autoimmune testing was also negative, including antinuclear antibody, extractable nuclear antigens (RNP, Smith), and a broad paraneoplastic antibody panel. Infectious studies were negative for herpes simplex virus, Lyme, syphilis (Venereal Disease Research Laboratory test), *Legionella*, and *Streptococcus pneumoniae*. Electrodiagnostic testing was performed on the second day of hospitalization after ICU transfer. Nerve conduction studies showed slowed median motor onset with small amplitudes and absent F-waves, while sensory studies were technically limited. Needle electromyography (EMG) demonstrated fibrillations and complex repetitive discharges in multiple muscles (extensor digitorum communis, tibialis anterior, rectus femoris, and medial gastrocnemius), with recruitment patterns suggestive of, but not diagnostic for, a myopathy. These findings argued against a primary neuromuscular junction disorder or demyelinating polyneuropathy and favored an acute myopathic process. ECG demonstrated sinus bradycardia with first-degree atrioventricular block and intraventricular conduction delay on one tracing and atrial fibrillation with possible anterior infarct of indeterminate age on a subsequent tracing. Despite these abnormalities, transthoracic echocardiography showed preserved biventricular size and function without wall motion abnormalities or pericardial effusion. Although troponin was elevated and ECGs showed atrial fibrillation with conduction delay, the patient did not report chest pain, palpitations, or heart failure symptoms. These findings did not provide clear support for myocarditis, though it could not be entirely excluded in the setting of ICI therapy without cardiac biopsy.

The differential diagnosis at that stage included an ICI-related neuromuscular complication, GBS, Miller Fisher variant (MFV), and influenza-associated encephalopathy (IAE). GBS and MFV were less likely given the absence of albuminocytologic dissociation in the cerebrospinal fluid and the lack of ophthalmoplegia. IAE was also considered but was not supported by preserved cognition, a normal brain MRI, and focal fatigable ocular-bulbar weakness. The combination of ocular-bulbar weakness, respiratory failure, elevated CK and aldolase, positive AChR antibody, and EMG findings consistent with a myopathic process most strongly supported an ICI-related irAE manifesting as myasthenia-myositis overlap syndrome. This entity falls within the broader MMM spectrum, in which not all patients exhibit all three features; however, even partial overlap is clinically significant. Although myocarditis was possible given troponin elevation and ECG changes, it was not supported by clinical symptoms or echocardiography.

Taken together, this constellation has been described after programmed death-1 or PD-L1 plus CTLA-4 therapy and requires urgent immunomodulation. High-dose IV methylprednisolone was initiated on hospital day 1 (250 mg every six hours, totaling 5 g over five days). On hospital day 2, IVIG was started at 0.4 g/kg/day and extended to a full five-day course (total 2 g/kg). Because neurological improvement remained limited after completion of IVIG, plasma exchange (PLEX) was initiated on hospital day 6 and performed every other day for a planned total of five sessions. After the third PLEX session, the patient was reliably following commands, left ptosis began to recede, and facial strength improved. However, horizontal gaze limitation persisted, consistent with the expected slower recovery of extraocular movements in immune-mediated myasthenic crises with concurrent myositis. Pyridostigmine was initially started at 60 mg via feeding tube three times daily and titrated up to 120 mg three times daily for symptomatic control but was later held due to concerns about secretion management. Durvalumab and tremelimumab were withheld, and neurology, oncology, and critical care teams coordinated daily reassessments of cranial nerve function, ventilator-weaning potential, and cardiac biomarkers.

After the third PLEX session, he was reliably following commands, left ptosis began to recede, and facial strength improved, though horizontal gaze remained limited. This trajectory was consistent with the expected slower recovery of extraocular movements in immune-mediated myasthenic crises complicated by inflammatory myopathy. The team planned to complete the PLEX series, continue careful respiratory monitoring, avoid further checkpoint dosing, and reassess the patient for longer-term immunosuppression after they had stabilized.

Despite these interventions, the patient experienced recurrent respiratory decline. After multidisciplinary discussion, including ethics and psychiatry consultation, he consistently expressed a desire to discontinue life-prolonging therapy. He was transitioned to inpatient hospice in the ICU so the family could remain at his bedside, and he was initiated on comfort-focused measures. He ultimately passed away peacefully with family present on hospital day 15, approximately 15 days after ICU admission and six weeks after receiving his first cycle of durvalumab plus tremelimumab.

## Discussion

Dual checkpoint blockade with single-dose tremelimumab plus durvalumab (the STRIDE regimen) has improved overall survival in unresectable HCC [1,2]. Our patient instead received repeated tremelimumab every four weeks in combination with durvalumab, a non-STRIDE regimen. This distinction is clinically relevant because the safety and efficacy profile reported in the HIMALAYA trial pertains to a single priming dose of tremelimumab with durvalumab maintenance. In contrast, continuous CTLA-4 exposure may plausibly increase the probability or severity of immune-related toxicity. Prospective safety data for repeated-dose tremelimumab in HCC remain limited. irAEs arise from the loss of immune tolerance after checkpoint blockade and may affect nearly any organ system. Although neurologic irAEs are relatively rare, they are often fulminant.

Fatal immune-related toxicities disproportionately include myocarditis and myositis, and MG is frequently observed among myocarditis deaths [7]. Durvalumab monotherapy has been linked in case reports to MMM overlap, indicating that PD-L1 blockade alone can precipitate the triad [8,9]. Qin et al. recently reported that ICI-related MG often presents within the first month of therapy and carries a worse prognosis compared to idiopathic MG [10]. Taken together, these findings support early recognition and escalation of care in patients with neuromuscular complaints after checkpoint inhibition. Our patient developed symptoms within four weeks of initiating therapy, which is consistent with this early time course.

MMM overlap represents one of the most severe ICI complications, with reported mortality approaching 38% [3]. Features such as ocular-bulbar weakness, early respiratory compromise, and concurrent CK/troponin elevations should immediately raise suspicion. In this case, AChR-binding antibody positivity, marked elevation of CK and aldolase, and elevated troponin levels strongly supported an overlap pathology. Electrodiagnostic testing further demonstrated fibrillations, complex repetitive discharges, and suspicious recruitment, providing objective evidence of a myopathic process. Although influenza A was detected, the fatigable MG phenotype and response to MG-directed therapy argue that viral infection served as a precipitant rather than the primary etiology. IAE was also considered, but the preserved cognition, absence of seizures, and normal MRI findings made this diagnosis unlikely. Notably, a normal echocardiogram does not exclude myocarditis, and ongoing surveillance with biomarkers and ECG remains essential [11]. In our patient, elevated troponin and conduction abnormalities raised suspicion for myocarditis, but the absence of clinical symptoms and preserved ventricular function meant myocarditis could not be confirmed. This case, therefore, illustrates the spectrum of MMM overlap, in which not all patients manifest all three features.

Guideline-concordant management includes discontinuation of ICI, high-dose corticosteroids, and early escalation to IVIG or PLEX for severe bulbar or respiratory involvement [5,6]. While our patient showed partial neurologic improvement, recurrent respiratory failure highlighted the limited efficacy of standard immunosuppression in overlap presentations. Observational and pooled data suggest better outcomes when IVIG or plasmapheresis are used early compared with steroids alone [4], although mortality remains substantial. Our patient’s course, with only modest improvement after IVIG and subsequent partial recovery with PLEX, demonstrates both the importance of timely escalation and the limitations of currently available therapy. For fulminant myocarditis, abatacept has been described in case reports as a rescue option [12], but robust data are lacking.

Finally, this case underscores the importance of early goals-of-care discussions in fulminant irAEs. Despite maximal immunotherapy, the patient chose to discontinue life-prolonging measures, a decision supported by psychiatry and ethics consultation. His transition to hospice illustrates the need to balance aggressive treatment with respect for patient autonomy in the setting of poor-prognosis immune toxicities. Clinicians should therefore maintain vigilance for MMM overlap, recognize that it exists on a spectrum, and integrate both aggressive immunotherapy and early goals-of-care planning into management.

## Conclusions

This case highlights the potential for fulminant myasthenia-myositis overlap with neuromuscular respiratory failure as an early and often fatal complication of dual PD-L1 plus CTLA-4 checkpoint blockade. Clinicians should maintain a high index of suspicion for ocular, bulbar, and respiratory symptoms in patients receiving immune checkpoint inhibitors, with prompt CK and troponin surveillance and early neuromuscular evaluation. Rapid escalation to IVIG or PLEX remains critical, though outcomes are frequently poor despite maximal immunotherapy. Ultimately, this case highlights the importance of multidisciplinary management, shared decision-making, and early discussions of care goals in striking a balance between aggressive treatment and patient-centered care in managing severe irAEs.
